# Modeling zero inflation is not necessary for spatial transcriptomics

**DOI:** 10.1186/s13059-022-02684-0

**Published:** 2022-05-18

**Authors:** Peiyao Zhao, Jiaqiang Zhu, Ying Ma, Xiang Zhou

**Affiliations:** 1 Department of Biostatistics, University of Michigan, Ann Arbor, MI 48109 USA; 2 Center for Statistical Genetics, University of Michigan, Ann Arbor, MI 48109 USA

**Keywords:** Spatial transcriptomics, Zero inflation, Overdispersion, Poisson model, Negative binomial model

## Abstract

**Background:**

Spatial transcriptomics are a set of new technologies that profile gene expression on tissues with spatial localization information. With technological advances, recent spatial transcriptomics data are often in the form of sparse counts with an excessive amount of zero values.

**Results:**

We perform a comprehensive analysis on 20 spatial transcriptomics datasets collected from 11 distinct technologies to characterize the distributional properties of the expression count data and understand the statistical nature of the zero values. Across datasets, we show that a substantial fraction of genes displays overdispersion and/or zero inflation that cannot be accounted for by a Poisson model, with genes displaying overdispersion substantially overlapped with genes displaying zero inflation. In addition, we find that either the Poisson or the negative binomial model is sufficient for modeling the majority of genes across most spatial transcriptomics technologies. We further show major sources of overdispersion and zero inflation in spatial transcriptomics including gene expression heterogeneity across tissue locations and spatial distribution of cell types. In particular, when we focus on a relatively homogeneous set of tissue locations or control for cell type compositions, the number of detected overdispersed and/or zero-inflated genes is substantially reduced, and a simple Poisson model is often sufficient to fit the gene expression data there.

**Conclusions:**

Our study provides the first comprehensive evidence that excessive zeros in spatial transcriptomics are not due to zero inflation, supporting the use of count models without a zero inflation component for modeling spatial transcriptomics.

**Supplementary Information:**

The online version contains supplementary material available at 10.1186/s13059-022-02684-0.

## Background

Spatially resolved transcriptomic studies perform gene expression profiling on many spatial locations of a tissue to characterize its transcriptomic landscape. Spatial transcriptomics studies are enabled by multiple spatial transcriptomics technologies that have emerged in the past few years. These spatial transcriptomic technologies include those that are based on single molecular fluorescent in situ hybridization (smFISH) [1–3] such as seqFISH, seqFISH+ [4, 5], and MERFISH [6, 7]; those that are based on microdissection techniques [8, 9] such as LCM [10] and tomo-seq [11]; and those that are based on in situ mRNA capturing followed by high-throughput sequencing techniques [12] such as spatial transcriptome (ST) [13], Slide-seq [14, 15], and high-definition spatial transcriptomics (HDST) [16]. Different spatial transcriptomic technologies measure different spatial units on the tissue with distinct spatial resolutions. For example, ST measures expression on multiple capture sites known as spots, each of which has a diameter of 100 μm and captures mRNA diffused from a neighborhood of likely 10–40 single cells [13]. The 10x Genomics technology has a spatial resolution of 55 μm with each spot assaying 1–10 cells (10x Genomics Space Ranger 1.1.0). Slide-seq has a spatial resolution of 10 μm with each measured location containing 1–3 cells [14, 15]. The high-definition spatial transcriptomics (HDST) has a spatial resolution of 2 μm [16]. Seq-Scope reaches sub-micrometer resolution and captures transcripts on locations with 0.5–0.8 μm distance apart from each other [17]. Laser capture microdissection sequencing (LCM-seq) is able to achieve single-cell resolution [10]. The smFISH-based technologies directly measure the transcript signals inside single cells via imaging and thus also reach single-cell resolution [1–3].

Regardless of the technology, the expression measurements obtained in spatial transcriptomics are often in the form of counts: they are collected either as the number of barcoded mRNA for any given transcript imaged in a single cell through smFISH-based techniques or as the number of sequencing reads mapped to any given gene through sequencing-based techniques. Consequently, many statistical methods developed for spatial transcriptomics analysis directly model the count data. For example, SPARK [18] models count data through an overdispersed Poisson distribution to detect genes that display spatial expression patterns. Stereoscope [19] and RCTD [20] models count data with negative binomial (NB) and Poisson regression, respectively, to perform cell type decomposition. gimVI [21] models count data with either NB or zero-inflated NB (ZINB) for missing gene expression imputation. Direct modeling of the count data in spatial transcriptomics can effectively account for the mean-variance relationship in the raw counts, thus achieving optimal analytic performance.

With the advance of spatial transcriptomics technologies, the count data collected from spatial transcriptomics has become increasingly sparse with a prevalence of zero values. For example, the recent seqFISH+ technology assays tens of thousands of genes through a number of wash-hybridization steps, with each mRNA being labeled by a sequence of hybridization signals that are captured by imaging. Due to imaging sensitivity and hybridization efficiency, the number of mRNA molecules measured in each cell by seqFISH+ can be low with many zero values. As another example, the sequencing-based technologies, limited by the total sequencing depth, also yield sparse count measurements especially as the number of measured spatial locations becomes increasingly large. With the prevalence of sparse counts and excessive zeros from recent spatial transcriptomics, one naturally wonders what types of count models one should use to describe these data and whether the excessive zeros observed in these data reflect technical artifacts or actual biological variation. Indeed, in the parallel field of single-cell RNA sequencing (scRNA-seq) studies, it has long been debated whether one should treat the zero values as missing data and use a zero-inflated model for their modeling [22–24] or whether the zeros belong to an integrated component of the count generating process and could be fully accounted for by a simple Poisson model or an overdispersed Poisson model such as a Poisson mixed model or a negative binomial (NB) model [25–27]. Examining and understanding the statistical properties of the excessive zero values in spatial transcriptomics is important, as it can help determine whether it is important to perform imputation for the zero values [28–31] and/or whether it is necessary to include a zero inflation component in statistical modeling, thus facilitating the development of best practices for various data analytic tasks in the field.

Here, we present a comprehensive analysis on 20 spatial transcriptomics datasets collected from 11 distinct technologies to characterize the distributional properties of the gene expression count data and understand the statistical property of the zero values. Specifically, for each data in turn, we carried out cross-gene analysis, gene-specific analysis, location-specific analysis and conditional analysis to characterize the goodness of fit for a range of count models on the gene expression counts and perform formal hypothesis tests to understand the overdispersion and zero inflation patterns of gene expression. Our study provides the first comprehensive evidence supporting the use of count models without a zero inflation component for modeling spatial transcriptomics, dovetailing the recent findings in the single-cell literature that modeling zero inflation is not necessary, at least for UMI-based technologies [25–27].

## Results

### Most spatial transcriptomics data contain an excessive amount of zero values and display overdispersion

We investigated the statistical distributional properties of the gene expression count data collected from different spatial transcriptomics technologies. To do so, we obtained a total of 20 spatial transcriptomics datasets from 11 distinct technologies. These technologies include MERFISH (*n* = 1 dataset), seqFISH (*n* = 1), seqFISH+ (*n* = 1), STARmap (*n* = 1), paired cell sequencing (*n* = 1), LCM-seq (*n* = 1), NICHE-seq (*n* = 1), Tomo-seq (*n* = 1), HDST (*n* = 1), Slide-seq (*n* = 1), Slide-seqV2 (*n* = 1), 10X Visium (*n* = 7), and Spatial Transcriptomics (*n* = 2). These technologies can be generally categorized into two categories: a category of smFISH-based spatial transcriptomics technologies that measure gene expression at the single-cell level (i.e. seqFISH, seqFISH+, MERFISH) and a category of sequencing-based spatial transcriptomics technologies that measure gene expression on tissue locations with various spatial resolutions (i.e. the remaining datasets). For each dataset, we obtained the gene expression measurements across tissue locations in the form of a count matrix. For sequencing-based technologies, each element of the count matrix represents the number of reads mapped to each gene on each spatial location. For smFISH-based technologies, each element of the count matrix represents the number of in situ hybridization signals detected for each targeted gene in each cell.

**Fig. 1:**
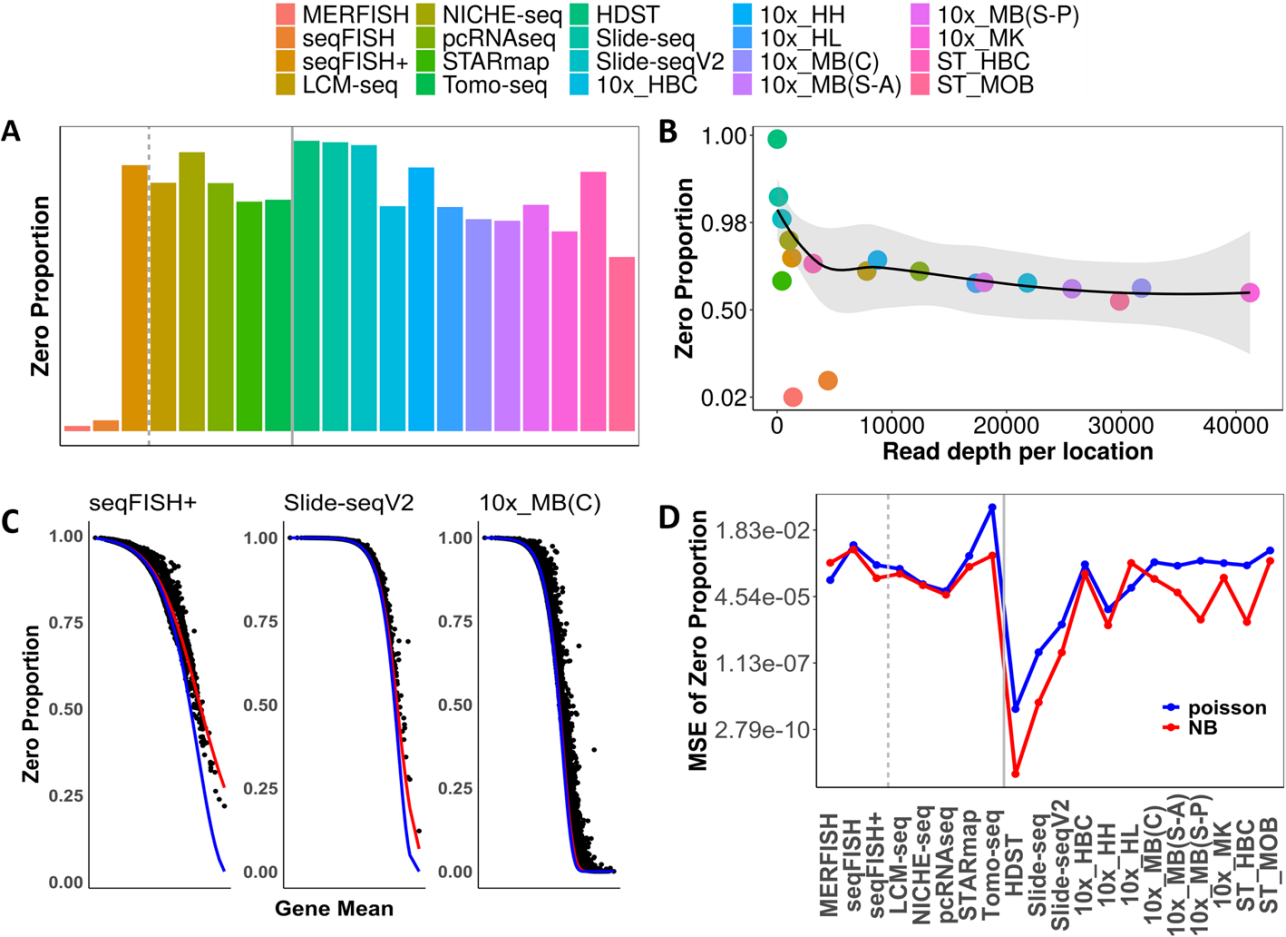
Most spatial transcriptomics datasets contain an excessive proportion of zero values. **A** The majority of spatial transcriptomics datasets (*x*-axis) contain a substantial proportion of zero values (*y*-axis). **B **The proportion of zero values in each dataset (y-axis; logit-transformed) is negatively correlated with the total counts per location (x-axis). Tomo-seq (zero proportion = 0.797; total count per location = 0.198 million) is an outlier and is not displayed on the panel. **C** The proportion of zero values for each gene (y-axis) is plotted against the expression mean (x-axis) for three example datasets that include seqFISH+, Slide-seqV2, and 10x_MB(C). The zero vs mean trend is fitted by either a Poisson model (blue line) or a negative binomial model (red line). **D** Mean square error (MSE) for the estimated zero proportion (*y*-axis; log transformed) based on either the Poisson model (blue line) or the negative binomial (red line) across datasets (*x*-axis). In both **A** and **D**, the gray dotted line separates smFISH-based technologies from the sequencing-based technologies and solid line separates single-cell resolution technologies to spot-level resolution technologies

The expression count matrix from various technologies, with the only exception of seqFISH and MERFISH, are in sparse forms, consisting of an excessive proportion of zeros (Additional file 1: Table S1). Specifically, while the proportion of zero counts in MERFISH and seqFISH are only 1.79% and 1.96% respectively, the proportion of zero counts in the other datasets ranges from 59.96% (for ST) to 99.96% (for HDST) with a mean of 75.85% (median = 79.34%; Fig. 1A). The proportion of zeros in each dataset is negatively correlated with the average total count per location in the dataset (Spearman correlation = −0.65; *p*-value = 0.002; Fig. 1B). (The total count per location is equivalent to the sequencing read depth per location, for sequencing-based spatial transcriptomics.) We further visualized the relationship between the estimated zero proportion and the estimated mean expression by plotting the two against each other across all genes (Fig 1C and Additional file 7: Fig. S1). We found that the gene-specific zero proportion is inversely related to the gene-specific expression mean. The inverse relationship between zero proportion and expression mean can be accounted for by a negative binomial (NB) distribution across the transcriptome (red line; Fig. 1C, D), and to a lesser extent, by a Poisson distribution (blue line; Fig. 1C, D).

**Fig. 2:**
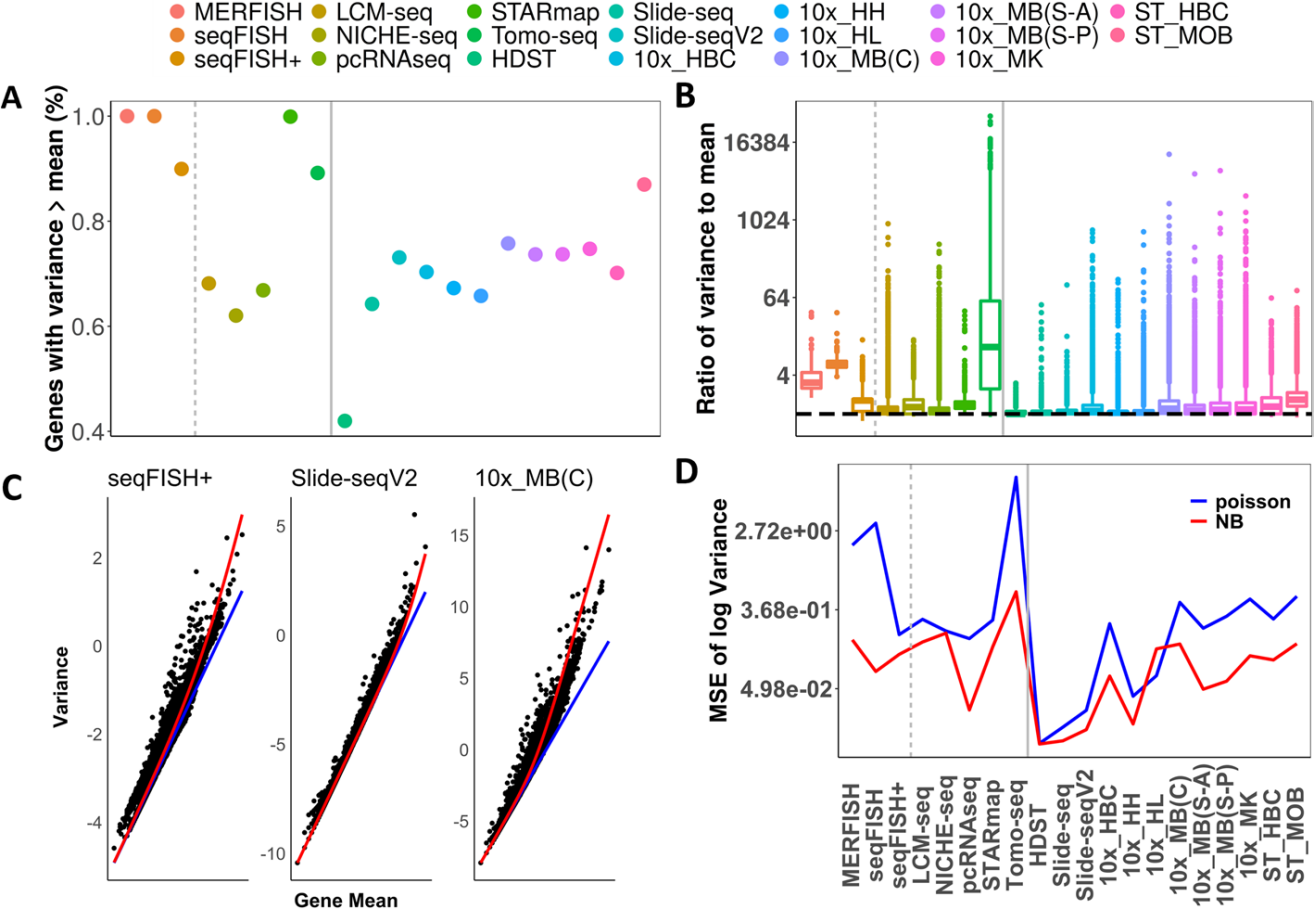
Most spatial transcriptomics datasets display overdispersion. **A** The proportion of genes with variance greater than mean (*y*-axis) across datasets (*x*-axis). **B** The median ratio of variance to mean (*y*-axis) across datasets (*x*-axis), where the horizontal line *y* = 1 shows where the variance equals the mean. **C** The log transformed variance for each gene (*y*-axis) is plotted against the expression mean for datasets from three techniques: seqFISH+, Slide-seqV2, and 10x_MB(C). **D** Mean square error (MSE) for the estimated log transformed variance (*y*-axis) based on either the Poisson model (blue line) or the negative binomial model (red line) across datasets (*x*-axis). In **A**, **B**, and **D**, the gray dotted line separates smFISH-based technologies from the sequencing-based technologies and solid line separates single-cell resolution technologies to spot-level resolution technologies

Besides zero inflation, the expression count matrix from the majority of the technologies also displays appreciable overdispersion. Specifically, in each dataset we calculated the proportion of genes that have a sample variance greater than the sample mean, as these genes display potential overdispersion that is not explained by a Poisson model. We found that the proportion of potentially overdispersed genes ranges from 41.98% (for HDST) to 100% (for MERFISH and seqFISH) across datasets, with an average estimate of 75.69% (median = 73.39%; Fig. 2A). For each dataset in turn, we contrasted the sample variance with the sample mean for every gene by calculating a ratio between the two and obtained the median ratio across all genes as a metric to quantify the extent of overdispersion in the dataset. We found that the median ratio ranges from 1.00 (for HDST) to 10.90 (for Tomo-seq) across datasets, with a median value of 1.17 (average = 2.00; Fig. 2B). We further visualized the relationship between the expression variance and mean by plotting the two against each other across genes (Fig. 2C and Additional file 7: Fig. S2). We found that the gene-specific variance is positively related to the gene-specific expression mean, with the relationship captured by a NB distribution (red line; Fig. 2C, D) but not a Poisson distribution (blue line; Fig. 2C, D).

### Poisson and negative binomial distributions are the preferred count models for most genes across most datasets

**Fig. 3:**
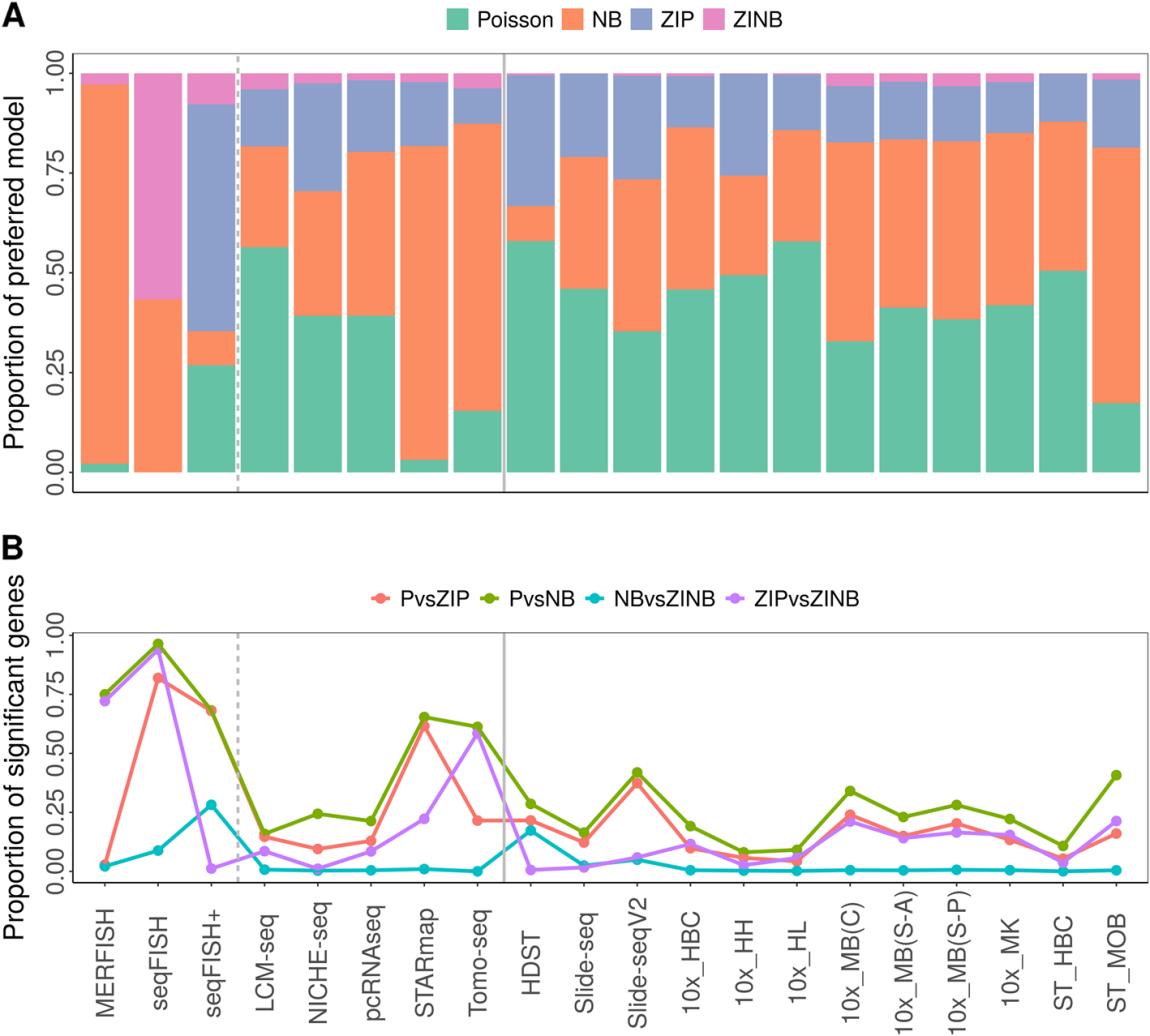
Gene-specific analysis, including model selection and likelihood ratio tests, across datasets. **A** The proportion of genes that prefer each of the four count models based on model selection with AIC (*y*-axis) is displayed for each dataset (*x*-axis). The four models include Poisson (Aquamarine), negative binomial (NB; Tangerine), zero-inflated Poisson (ZIP; Polo Blue), and zero-inflated negative binomial (ZINB; pink). The Poisson model and the negative binomial model are the preferred models across datasets. **B** The proportion of genes that are significant in each of the four likelihood ratio tests (*y*-axis) is displayed for each dataset (*x*-axis). The four LRT tests include the test on P vs ZIP (salmon), P vs NB (green), NB vs ZINB (cyan), and ZIP vs ZINB (purple)

We carefully examined the expression count distribution for each gene separately. To do so, for each gene in turn, we fitted its expression levels across tissue locations using four distinct count models. The four models include a Poisson model, a NB model, a zero-inflated Poisson (ZIP) model, and a zero-inflated negative binomial (ZINB) model. Based on these models, we carried out analyses to evaluate the goodness-of-fit of the four count models on fitting the expression count data for one gene at a time. Specifically, we performed model selection for every gene to identify the model that best describes the gene expression pattern using the Akaike information criterion (AIC). In the analysis, in all datasets except for seqFISH and seqFISH+, we found that a large proportion of genes (median = 82.23%; range from 66.74% for HDST to 97.14% for MERFISH) prefers either the Poisson model (median = 41.60%) or the NB model (median = 40.84%; Fig. 3A). The only exceptions are the datasets with smFISH technology: the proportion of genes preferring the Poisson model is low for both seqFISH (0.00%) and MERFISH (2.14%), while the proportion of genes preferring the NB distribution is low for seqFISH+ (8.52%) but high in MERFISH (95%). For most datasets, except for seqFISH and seqFISH+, only a small proportion of genes prefer the ZIP model (median = 14.37%), and even fewer prefer the ZINB model (median = 1.95%). However, a large proportion of genes prefer ZIP in seqFISH+ (56.84%), while a large proportion of genes prefer ZINB in seqFISH (56.63%; Fig. 3A). Overall, the results suggest that the Poisson and NB models are preferred for modeling the majority of genes across the majority of datasets.

### Modeling overdispersion generally accounts for zero inflation, but not vice versa

We performed gene-specific analysis to characterize the zero inflation and overdispersion patterns for every gene. First, for each data in turn, we performed two sets of likelihood ratio tests (LRT) to formally identify overdispersed genes and zero-inflated genes. The two sets of LRT examine whether the gene of focus displays significant zero inflation (P vs. ZIP) and/or overdispersion (P vs. NB) on top of the Poisson distribution. In the first LRT (P vs. ZIP), we found that an appreciable proportion of genes display zero inflation not accounted for by the Poisson distribution. Such proportion ranges from 2.86% (for MERFISH) to 81.93% (for seqFISH), with a mean of 22.91% across datasets (median = 14.84%; Fig. 3B and Additional file 2: Table S2). In the second LRT (P vs. NB), we also found that an appreciable proportion of genes display overdispersion not accounted for by the Poisson distribution. Such proportion ranges from 8.10% (for 10x_HH) to 96.39% (for seqFISH), with a mean of 35.51% across datasets (median = 26.27%; Fig. 3B and Additional file 2: Table S2). Importantly, the overdispersed genes detected by the second LRT have a substantial overlap with the zero-inflated genes detected by the first LRT. Specifically, the vast majority of the zero-inflated genes display overdispersion (ranges from 71.64% for NICHE-seq to 100% for MERFISH and seqFISH; mean proportion = 95.43%; median proportion = 98.87%). A substantial proportion of overdispersed genes also display zero inflation (ranges from 27.87% for seqFISH to 99.56% for seqFISH+; mean proportion = 64.25%; median proportion = 64.58%; not include MERFISH which has only four zero-inflated genes) (Fig. 3B and Additional file 2: Table S2).

The relatively high overlap between the overdispersed and zero-inflated genes prompted us to investigate formally the relationship between zero inflation and overdispersion. To do so, we carried out two additional LRT to examine whether the observed zero inflation can be explained by overdispersion (via a third LRT on NB vs. ZINB) and whether the observed overdispersion can be explained by zero inflation (via a fourth LRT on ZIP vs. ZINB). The third LRT allows us to examine whether a zero inflation component is needed on top of an NB distribution to characterize the excessive zeros in spatial transcriptomics, or whether the modeling of overdispersion by NB is sufficient to account for zero inflation. In the analysis, we found that, for most spatial transcriptomics technologies, with two exceptions (seqFISH+ and HDST), only a very small proportion of genes would benefit from an additional zero inflation component on top of the NB model: the proportion of significant genes in the third LRT ranges from 0.03% (for Tomo-seq) to 8.84% (for seqFISH), with a mean of 1.37% (median = 0.51%) across datasets. Only the seqFISH+ and HDST data can benefit from explicit modeling of zero inflation on top of the NB model: 28.22% and 17.31% genes are significant in the third LRT for the two datasets, respectively. The fourth LRT, on the other hand, allows us to examine whether an overdispersion component is needed on top of a ZIP distribution to characterize the overdispersion in spatial transcriptomics, or whether modeling of zero inflation by ZIP is sufficient to account for the overdispersion. In the analysis, we found that the proportion of genes would benefit from an additional overdispersion on top of the ZIP model ranges widely depending on datasets. For example, while a small proportion of genes in the HDST (0.60%), Slide-seq (1.67%), or Human Heart from 10x Visium data (2.64%) could benefit from modeling overdispersion, a substantial proportion of genes in Mouse Brain (Coronal) from 10x Visium (21.13%), STARmap (22.25%), and Tomo-seq (58.46%) could benefit from modeling overdispersion. Such proportion appears to be dependent on the number of genes preferring NB model based on AIC (Spearman correlation = 0.9038, *p*-value = 1.82e−06). The third and fourth sets of LRTs suggest that the observed overdispersion in the majority of the spatial transcriptomics datasets is not due to zero inflation, but not vice versa.

### Overdispersion and zero inflation are primarily due to expression heterogeneity and the spatial distribution of cell types across tissue locations

One potential source that may contribute to the observed overdispersion and/or zero inflation is the gene expression heterogeneity across tissue locations. Here, we examined the extent to which the observed over dispersion and/or zero inflation can be accounted for by such expression heterogeneity across locations. To do so, we first categorize tissue locations into distinct location clusters either based on the original study or by performing clustering analysis (details in “Methods”). For spatial transcriptomics with single-cell resolution, such clustering information effectively contains cell type annotation information for the measured cells. For spatial transcriptomics with regional resolutions, such clustering information may contain tissue structure information for the measured locations. In either case, clustering on locations allows us to segregate tissue locations into location clusters, each containing a set of relatively homogeneous tissue locations or cells. With location clustering information, we performed a similar set of analyses as in the previous two sections to characterize the distributional properties of the expression count data in each location cluster separately. Such cluster-specific analyses allow us to effectively control for expression heterogeneity across locations and to focus the analysis on a set of locations that are relatively homogeneous.

**Fig. 4:**
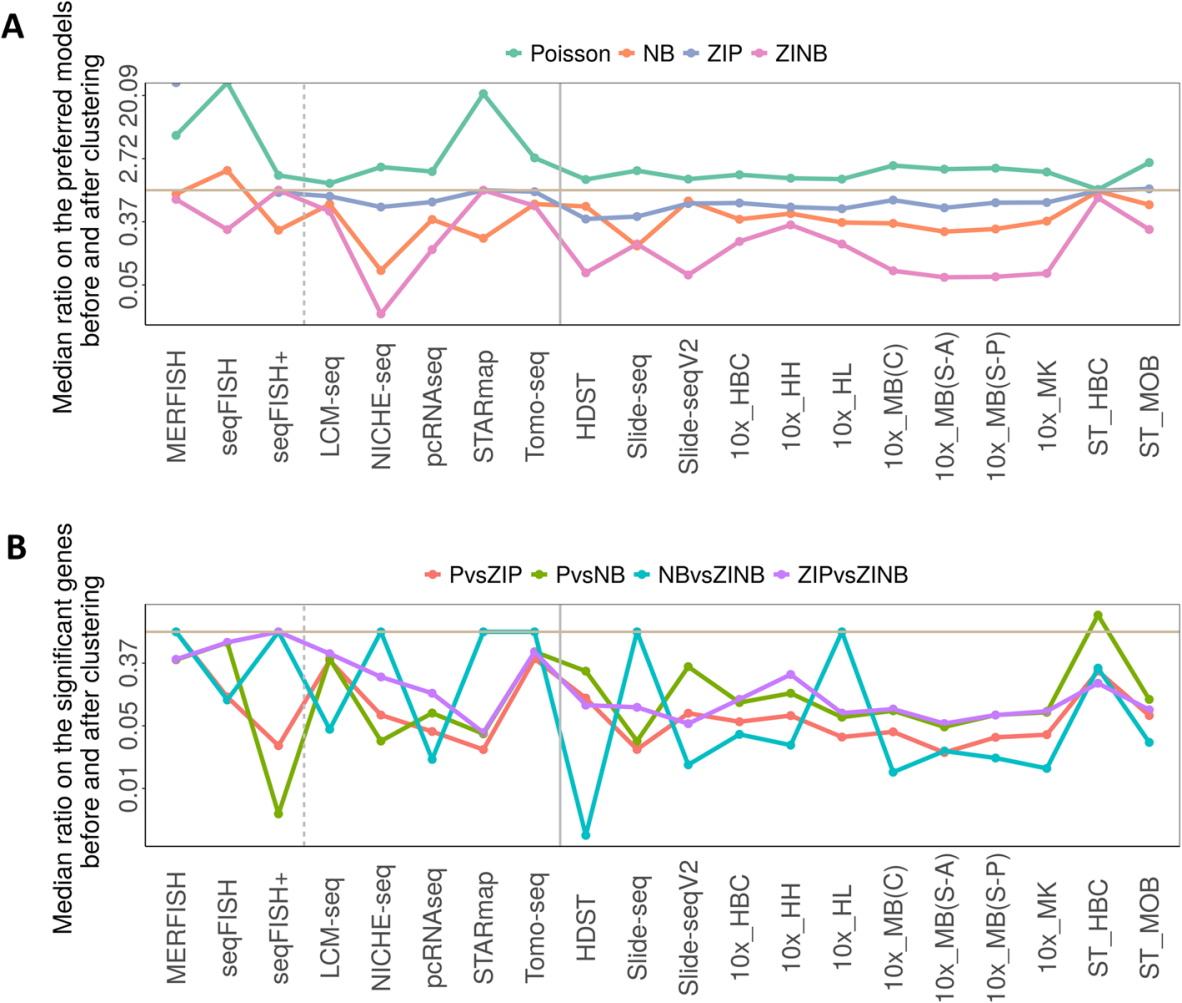
Location cluster-specific analysis reveals shifted count model preference and substantially reduced significant genes in likelihood ratio tests across datasets. **A** A ratio is computed in each cluster-specific analysis in each dataset to contrast the proportion of genes that prefer each of the four count models based on AIC after clustering to that before clustering. The median ratio across location clusters (*y*-axis; log-scale) is plotted for each dataset (*x*-axis). The four count models include Poisson (green), negative binomial (NB; salmon), zero-inflated Poisson (ZIP; cyan), and zero-inflated negative binomial (ZINB; purple). The gray horizontal line denotes a ratio value of one. The median ratio for the Poisson model is above one while the median ratio for the other models is below one in almost all datasets. A few data points are not displayed: the seqFISH data contains no gene preferring the ZIP model before clustering and after clustering; and the MERFISH data contains no gene preferring the ZIP model before clustering and an average of 1.43% of genes preferring ZIP model. **B** A ratio is computed in each location cluster in each dataset to contrast the proportion of significant genes in each the four LRT tests after clustering to that before clustering. The median ratio across location clusters (*y*-axis; log-scale) is plotted for each dataset (*x*-axis). The gray horizontal line denotes a ratio value of one. The four LRT tests include the test on P vs ZIP (Aquamarine), P vs NB (Tangerine), NB vs ZINB (Polo Blue), and ZIP vs ZINB (pink). The significant genes are substantially reduced after clustering analysis in most datasets

In the location cluster-specific analysis, we found that a substantial fraction of genes prefers the Poisson model over the other models (Fig. 4A). Specifically, apart from the seqFISH data, compared to the whole tissue analysis, the fraction of genes preferring the Poisson model in the cluster-specific analysis on average increases by 1.07 fold (for ST_HBC) to 20.90 fold (for STARmap), with a median increase of 1.79 fold across datasets. For example, in the human breast cancer dataset from 10x Visium, the proportion of genes that prefer the Poisson, NB, ZIP, and ZINB models is 45.84%, 40.65%, 12.81%, and 0.71%, respectively. In the cluster-specific analysis in the largest cluster, the proportion of genes that prefer the Poisson, NB, ZIP, and ZINB models becomes 68.99%, 20.44%, 10.42%, and 0.01%, respectively. The only dataset that was hard to quantify through fold change is seqFISH, where only a small proportion of genes prefer the Poisson model before (0%) and after (2.4–3.2%) location clustering.

We further examined the proportion of significant zero-inflated and overdispersed genes, as defined by the first LRT (P vs ZIP) and the second LRT (P vs NB), respectively. We found that the proportion of genes showing significant zero inflation or overdispersion substantially reduced in the cluster-specific analysis (Fig. 4B). Specifically, the proportion of zero-inflated genes in the cluster-specific analysis is on average only 3.19% (for 10x_MB(S-A) dataset) to 42.32% (for Tomo-seq dataset) as compared to the whole tissue analysis, with a median of 7.58%. Similarly, except for the ST_HBC data, the proportion of overdispersed genes in the cluster-specific analysis is on average only 3.47% (for seqFISH+ dataset) to 70.83% (for seqFISH dataset) as compared to the whole tissue analysis, with a median of 10.90%. For ST_HBC data, the proportion of overdispersed genes in the cluster-specific analysis increased to an average of 224% as compared to that of the whole tissue analysis. In addition, the significant genes from the other two LRTs are also substantially reduced in the cluster-specific analysis (Fig. 4B). The results suggest that a large fraction of genes in the majority of datasets are no longer zero-inflated or overdispersed after accounting for expression heterogeneity across location clusters.

Finally, we note that one important source of gene expression heterogeneity across tissue locations is the spatial distribution of cell types and the resulting cell type heterogeneity across locations. To examine whether the spatial distribution of cell types may contribute to the observed overdispersion and/or zero inflation, we performed conditional analyses. Specifically, we focused on nine spatial transcriptomics datasets where we were able to obtain a published single-cell RNA sequencing reference data measured on the same tissue (Additional file 3: Table S3). On each of the nine datasets, we performed reference-based cell type deconvolution using CARD [32] to infer the cell type composition on each tissue location. Afterwards, we carried out conditional analysis where we controlled for the estimated cell type compositions by including them in the count models as covariates (details in “Methods”). In the analysis, we found that the fraction of genes preferring the Poisson model after controlling for cell type compositions on average increases by 1.01 fold (for 10x_HH) to 1.88 fold (for 10x_MB(C)), with a median increase of 1.22 fold across datasets (Additional file 7: Fig. S3). In addition, the proportion of overdispersed genes after controlling for cell type compositions substantially reduced and is on average only 27.71% (10x_MB(S-P)) to 91.16% (10x_HH) of that before, with a median of 53.53%. However, the proportion of zero-inflated genes after controlling for cell type compositions may increase or decrease depending on the dataset: for example, such proportion increases 1.65 fold for 10x_HH but decreases to 52% for 10x_HBC after controlling for cell type compositions. Overall, the conditional analysis results suggest that the degree of overdispersion substantially decreases after controlling for cell type compositions, highlighting the importance of the spatial distribution of cell types in contributing to the observed over dispersion.

## Discussion

We have presented a comprehensive analysis for characterizing the distributional properties of the count data collected from multiple spatial transcriptomics technologies. We have demonstrated that a substantial fraction of genes displays overdispersion or zero inflation that cannot be accounted for by a simple Poisson model. We found that zero inflation and overdispersion are different terms that potentially capture similar features of the data, as many zero-inflated genes are overlapped with overdispersed genes. In addition, we found that zero inflation generally can be accounted for by modeling overdispersion while overdispersion often cannot be accounted for by modeling zero inflation. Consequently, we show that the Poisson model and the NB model, which accounts for overdispersion on top of the Poisson model, are sufficient for modeling the majority of genes across most spatial transcriptomics technologies. Importantly, we show that a major source of overdispersion and zero inflation observed in spatial transcriptomics is gene expression heterogeneity across tissue locations partially caused by the spatial distribution of cell types. Indeed, when we control for cell type compositions or focus on a relatively homogeneous set of tissue locations, which represents the same cell type or locations that consists of a similar composition of cell types, the number of overdispersed and/or zero-inflated genes is substantially reduced. In the cluster-specific analyses and cell type conditional analyses, a simple Poisson model is often sufficient to fit the gene expression data.

Our results suggest that the excessive zero values observed in spatial transcriptomics can be accounted for by direct modeling of overdispersion without introducing an extra zero inflation term. Consequently, the zero values in spatial transcriptomics potentially reflect biological variation and unlikely represent technical artifacts. Therefore, just like in scRNA-seq studies with UMI data, imputation of zero values in spatial transcriptomics may not be desirable and may induce unwanted noise and negatively impact downstream analysis [25–27]. In addition, because the major source of overdispersion and zero inflation is expressing heterogeneity across locations partially caused by the spatial distribution of cell types, direct testing of overdispersion and/or zero inflation in each location cluster could help determine the level of heterogeneity in the cluster, thus facilitating the determination of the optimal number of clusters in the data. A recent study in scRNA-seq proposes the HIPPO framework to leverage excessive zeros on top of the Poisson model to explain cellular heterogeneity and determine the number of cell type clusters [25]. Adaption of similar approaches from scRNA-seq studies to spatial transcriptomics will likely improve spatial clustering analysis and facilitate various downstream analysis such as the identification of spatially expressed genes [18, 33, 34].

As a side note, we have included the total count per location as an offset term in the mean component of each of the four count models following that of [18, 20, 22]. Due to variation of capture efficiency or imaging quality, the total count per location can vary quite substantially across tissue locations. Consequently, including total count per location accounts for the variability across tissue locations and often improves the fitting of different count models for the majority of datasets (Additional file 7: Fig. S4). Therefore, explicit adjustment for total count per location is also recommended for modeling spatial transcriptomics dataset.

## Conclusion

In conclusion, our results suggest that the excessive zeros in spatial transcriptomics are not due to zero inflation and that it is not necessary to include a zero inflation component for the statistical modeling of gene expression counts from spatial transcriptomics. Instead, the Poisson model and the overdispersed Poisson models such as the negative binomial model are often sufficient for modeling the majority of genes across most spatial transcriptomics technologies.

## Methods

### Spatial transcriptomics datasets

We examined a total of 20 spatial transcriptomics datasets collected from 11 different technologies listed in detail below.

#### Spatial transcriptomics (two datasets)

Spatial transcriptomics (ST) is a technology that allows for the visualization and quantitative analysis of the transcriptome with spatial resolution on individual tissue sections [13]. ST places tissue sections on glass slides with arrayed oligonucleotides containing positional barcodes placed on locations known as spots. Each spot is of 100 μm in diameter, with a 200-μm center to center distance placed between spots. We downloaded the mouse olfactory bulb data and the human breast cancer data [13] from the Spatial Research lab (https://www.spatialresearch.org/resources-published-datasets/doi-10-1126science-aaf2403/). We used files “MOB Replicate 11” (denoted as ST_MOB for mouse olfactory bulb) and “Breast Cancer Layer 2” (denoted as ST_HBC for human breast cancer), which contain 16,218 and 14,789 genes measured on 262 and 251 spatial locations, respectively. After removing the genes that are not expressed on any spots, we analyze the final datasets that contain 16,218 and 14,789 genes.

#### 10X Visium (seven datasets)

The 10x Genomics Visium is a platform that builds on the foundation of the Spatial Transcriptomics technique, with improvement on resolution, scale, and workflow (10x Genomics Space Ranger 1.1.0). In 10x Visium technology, each spot/location is of 55 μm in diameter, with a 100-μm center to center distance allocated between spots. We downloaded seven visium datasets from the 10X Visium spatial gene expression repository under item Visium Spatial Gene Expression 1.1.0 (https://www.10xgenomics.com/resources/datasets/). The seven datasets include the human breast cancer data [35] (denoted as 10x_HBC, block A section 1; with 36,601 genes, 3798 locations, and 24,923 analyzed genes after removing genes that are not expressed on any spots), the human heart data [36] (denoted as 10x_HH; 36,601 genes, 4247 locations, and 20,917 analyzed genes), the human lymph node data [37] (denoted as 10x_HL, 36,601 genes, 4035 locations, and 25,187 analyzed genes), the mouse kidney coronal section data [38] (denoted as 10x_MK; 32,285 genes, 1438 locations, and 20,100 analyzed genes), the mouse brain coronal section data [39] (denoted as 10x_MB(C); 32,285 genes, 2702 locations, and 21,949 analyzed genes), the mouse brain sagittal-posterior data [40] (denoted as 10x_MB(S-P); 32,285 genes, 3355 locations, and 21,334 analyzed genes), and the mouse brain sagittal-anterior data [41] (denoted as 10x_MB(S-A); 32,285 genes, 2695 locations, and 21,363 analyzed genes).

#### Slide-seq (one dataset)

Slide-seq is a technology that infers RNA locations by sequencing [14]. This method transfers RNA from tissue sections onto a surface covered in uniquely DNA-barcoded 10-μm microparticles (“beads”) with known positions. It enables spatially resolved gene expression data at resolutions comparable to the sizes of individual cells. We downloaded the mouse cerebellum data [14] from Broad institute’s single-cell repository (https://singlecell.broadinstitute.org/single_cell) with ID SCP354. We obtained the section “Puck_180430_6,” which contains 18,671 genes and 25,551 locations. After removing the genes that are not expressed on any spots, we analyzed the final dataset that contains 17,754 genes.

#### Slide-seqV2 (one dataset)

Slide-seqV2 is a technology that makes modifications on library generation, bead synthesis, and array indexing to markedly improve the mRNA capture sensitivity of the original Slide-seq technology [15]. We downloaded the mouse brain Slide-seqV2 dataset [15] from the Broad institute’s single-cell repository with ID SCP815. We obtained the section “Puck_190921_19,” which contains 22,683 genes and 33,611 locations. After removing the genes that are not expressed on any spots, we analyzed the final dataset that contains 21,625 genes.

#### High-definition spatial transcriptomics (one dataset)

High-definition spatial transcriptomics (HDST) is a technology that captures RNA from histological tissue sections on a high-resolution (2 μm) and high-density spatially barcoded bead array [16]. We downloaded the HDST dataset collected on the mouse olfactory bulb [16] from the Broad institute’s single-cell repository with ID SCP420. We used the file “CN24_D1,” which contains 19,950 genes and 181,367 locations. All genes in the data are expressed on at least one location.

#### STARmap (one dataset)

Spatially resolved transcript amplicon readout mapping (STARmap) is a technology that allows for three-dimensional (3D) intact-tissue RNA sequencing by integrating hydrogel-tissue chemistry, targeted signal amplification, and in situ sequencing at single-cell resolution [42]. We downloaded the data collected on mouse visual cortex [42] from STARmap repository (https://www.dropbox.com/sh/f7ebheru1lbz91s/AABYSSjSTppBmVmWl2H4s_K-a?dl=0), which contains 1020 genes and 1549 locations/cells. All genes in the data are expressed on at least one location.

#### LCM-seq (one dataset)

Laser capture microdissection sequencing (LCM-seq) is a technology that characterizes the transcriptomics on laser capture micro-dissected villus segments and subsequent spatial transcriptomics reconstruction [43]. We downloaded data collected on mouse jejunum [43] from zenodo (https://zenodo.org/record/1320734), which contains 27,998 genes and 1383 locations. After removing the genes that are not expressed on any spots, we analyzed the final dataset that contains 14,220 genes.

#### NICHE-seq (one dataset)

The NICHE-seq is a technology that combines photoactivatable fluorescent markers, two-photon laser scanning microscopy (TPLSM), and flow cytometry–based fluorescence-activated cell sorting (FACS) coupled to massively parallel single-cell RNA-Seq (MARS-Seq) [44]. They further perform tissue dissociation by utilizing transgenic mice’s expressing a photoactivatable green fluorescent protein (PA-GFP) to get the spatial information. We downloaded data collected on mice immune niches [44] from Gene Expression Omnibus repository with ID GSE104054, specific file GSM2788364_AB1655.txt.gz, which contains 34,016 genes and 384 locations. After removing the genes that are not expressed on any spots, we analyzed the final dataset that contains 11,364 genes.

#### Tomo-seq (one dataset)

Tomo-seq is a technology that retains spatial information by cryo-sectioning the tissue sample along a specific axis and then performing RNA sequencing on the individual sections [11]. We obtained data collected on male *C. elegans* [11] from Gene Expression Omnibus repository with ID GSE114723, which includes 16,869 genes and 96 locations. All genes are expressed on at least one location.

#### Paired cell sequencing (one dataset)

Paired cell sequencing (NICHE-seq) is a technology that utilized massively parallel single-cell RNA-Seq (MARS-Seq) to sequence pairs of hepatocytes and adjacent endothelial cells [45]. They later used smFISH to obtain zonation profile to determine the tissue location, and thus get the zonation patterns of endothelial genes. We downloaded data on liver endothelial cells (LECs) [45] from Gene Expression Omnibus repository with ID GSE108561, which contains 33,948 genes and 4621 locations. After removing the genes that are not expressed on any spots, we analyzed the final dataset that contains 19,023 genes.

#### seqFISH (one datasets)

Sequential fluorescence in situ hybridization (seqFISH) is a technology that enables the identification of thousands of RNA transcripts directly in single cells with their spatial context preserved by labeling transcripts with fluorescent probes in sequential rounds of hybridization [5]. We downloaded expression data from [5] and extracted cell counts from the region annotated as number 43 containing 249 genes and 257 cells. All genes are expressed in at least one cell.

#### seqFISH+ (one dataset)

SeqFISH+ improves the previous seqFISH technique with higher accuracy and sub-diffraction-limit resolution [4]. We downloaded the olfactory bulb of the mouse brain [4] from Cai’s lab (https://github.com/CaiGroup/seqFISH-PLUS). We analyzed the olfactory bulb data, which contains 10,000 genes and 2050 locations. All genes are expressed in at least one cell.

#### MERFISH (one dataset)

Multiplexed error-robust fluorescence in situ hybridization (MERFISH) is the technology that allows imaging individual RNA molecules by performing imaging-based cell type identification paired with multiplexed in situ hybridization [34]. We downloaded data collected from the mouse hypothalamic preoptic region [34] from github (https://github.com/Teichlab/SpatialDE/blob/master/Analysis/MERFISH/data/rep6/middle_exp_mat.csv). The data contains 140 genes measured on 1056 locations. All genes are expressed in at least one cell.

### Cross-gene and gene-specific analysis

For each dataset in turn, we extracted the gene expression count matrix and removed genes with zero counts on all spatial locations. We then performed multiple analyses described as follows.

First, we computed for each gene three summary statistics: mean, variance, and proportion of zeros. We visualized the relationship between the variance and the mean as well as the relationship between the zero proportion and the mean, across all genes. In addition, we fitted these two relationships across all genes using a cross-gene Poisson model or a cross-gene negative binomial (NB) model. Specifically, the relationship between the variance (*σ*^2^) and the mean (*μ*) is described as *σ*^2^ = *μ* by the cross-gene Poisson model and is described as *σ*^2^ = *μ* + *μ*^2^/*ϕ* by the cross-gene NB model, where *ϕ* is the overdispersion parameter. The relationship between the proportion of zero (*p*) and the mean (*μ*) is described as *p* = e^−μ^ by the cross-gene Poisson model and is described as *p* = (1 + *ϕ*/*μ*)^−*ϕ*^ by the cross-gene NB model. We used the nonlinear least squares technique implemented in the nls function in R [46] to fit the cross-gene NB model, where we used the default Gauss-Newton algorithm to obtain the dispersion parameter *ϕ*. We then obtained the estimated variance and zero proportion based on the mean using either the cross-gene Poisson or the cross-gene NB model. We evaluated the estimation accuracy by computing the mean square error (MSE) based on either the zero proportion or the log transformed variance.

Second, we examined one gene at a time and fitted expression data using four count models. The four count models include Poisson, NB, zero-inflated Poisson (ZIP), and zero-inflated negative binomial (ZINB). We used the R package MASS [47] to fit Poisson and NB and used the R package pscl [48, 49] to fit ZIP and ZINB. For each gene in turn, we obtained the estimated mean, variance, and zero proportion from each of the four models according to the formula in Additional file 4: Table S4. During model fitting, we included the total read depth on each spatial location as an offset term in the mean component of different models. In addition, we relied on sparse matrix operations and used parallel computing functions in the R package Matrix to improve computation efficiency and reduce memory usage. Note that the NB model failed to converge in an appreciable fraction of genes (mean = 24.84%, median = 27.81% across datasets), because many of these genes (mean = 77.63%, median = 87.24%) have an estimated variance below the estimated mean. For genes where the likelihood function of the NB model failed to fit, we used the generalized estimating equations (GEE) method implemented in the gee R package with an identity correlation structure to estimate the mean and variance parameters for the NB model. With the mean and variance estimates from GEE, we obtained the dispersion parameter *ϕ* for every gene using the formula ${\hat{\sigma}}^2=\hat{\mu}+{\hat{\mu}}^2/\phi$, where ${\hat{\sigma}}^2$ is the estimated variance and $\hat{\mu}$ is the estimated mean. We plugged in the mean and dispersion estimates from GEE into the likelihood function of the NB model and obtained AIC. We then compared the AIC of the NB model using the GEE estimates with that using the estimates from the un-converged NB model. We chose the estimates that yielded the smaller AIC as the final estimates for the NB model.

Third, we performed formal hypothesis tests for each gene in turn to examine whether the gene of focus displays zero inflation and whether it displays overdispersion. Specifically, we carried out four sets of likelihood ratio tests using the R package lmtest [50]. The first test contrasts a Poisson model with a zero-inflated Poisson model (ZIP) and examines whether the gene of focus displays zero inflation that is not accounted for by a Poisson model. The second test contrasts a Poisson model with a negative binomial model (NB) and examines whether the gene displays overdispersion that is not accounted for by a Poisson model. The third test contrasts a NB model with a zero-inflated negative binomial model (ZINB) and examines whether the gene displays zero inflation that is not accounted for by a NB model. The fourth test contrasts a zero-inflated Poisson model (ZIP) and a zero-inflated negative binomial model (ZINB) and examines whether an overdispersion component is needed on top of modeling zero inflation. For all these tests, we declared significance based on a Bonferroni corrected *p*-value threshold of 0.05 that adjusted for the number of genes tested in each dataset. In addition, we examined the overlap between two gene sets—genes with significant overdispersion and genes with significant zero inflation—by calculating the proportion of the former that are also identified as the latter, and the proportion of the latter that are also identified as the former. Finally, besides formal hypothesis tests, we also calculated Akaike information criterion (AIC) for each count model and selected the best model with the lowest AIC as the preferred model for the gene of focus.

### Location clusters and cluster-specific analysis

We examined whether the observed zero inflation and/or overdispersion can be accounted for by gene expression heterogeneity across locations. To do so, we categorized the tissue locations in each dataset into different clusters either directly using the clustering labels from the original study (for five datasets: seqFISH+, STARmap, LCM-seq, Slide-seq, and HDST), or, in the absence of such information, by performing our own clustering analysis on the tissue locations (for the remaining 15 datasets). For our own clustering analyses in each of the 15 datasets, we followed a standard procedure recommended by Seurat [51]. Specifically, we used NormalizeData function with default setting to normalize the gene expression measurement on each location, where the function divided the read count on the location by the total counts there, multiplied by a scale factor of 10,000, and performed a log transformation afterwards (by adding a pseudo-count of 1 to avoid log transforming zero values). We obtained the top 2000 highly variable genes using the FindVariableFeatures function in the variance stabilizing transformation procedure (vst) in Seurat. We then extracted the top 15 principal components from the highly variable genes and performed Louvain clustering with the resolution parameter set to be 0.5. The clustering results for these data are shown in Additional file 7: Fig. S5 with detailed clustering information provided in Additional file 5: Table S5.

Clustering tissue locations effectively group together tissue locations that have similar gene expression profiles. In particular, for spatial transcriptomics with single-cell resolutions, clustering tissue locations is equivalent to clustering these locations/cells into distinct cell types. For spatial transcriptomics with regional resolutions, such clustering effectively allocates tissue locations into distinct tissue domains or into groups of locations that contains similar cell type compositions. With location clustering information, we performed the same sets of analyses described in the previous section, but in a cluster-specific fashion. Specifically, we performed model comparison by examining the AICs of the four count models for each gene in each cluster. We performed hypothesis tests for every gene in each cluster to contrast the Poisson model with either the NB model or the ZIP model. Such cluster-specific analyses characterize the distributional property of the expression count data in each location cluster separately, potentially removing the expression heterogeneity due to the spatial localization pattern of cell types and the expression variation across location clusters.

Finally, we examined the extent to which the observed zero inflation and/or overdispersion can be accounted for by the spatial distribution of cell types across tissue locations. To do so, we focused on nine spatial transcriptomics datasets where we were able to identify a corresponding single-cell RNA sequencing reference data [52–56] measured on the same tissue. The nine datasets include Slide-seq, 10x Visium (human breast cancer, mouse brain coronal section, mouse brain sagittal-anterior data, mouse brain sagittal-posterior, mouse kidney coronal section), and spatial transcriptomics (human breast cancer and mouse olfactory bulb) (Additional file 6: Table S6). For each dataset in turn, we performed reference-based cell type deconvolution using CARD [32] to infer the cell type composition on each tissue location. Afterwards, we treated the estimated cell type compositions as covariates in the count models and carried out the same set of analysis described in the previous section. Specifically, for each gene in turn, we performed model comparison by examining the AICs of the four count models and performed hypothesis tests based on the aforementioned four LRTs. These analyses allow us to characterize the distributional property of the expression count data while controlling for cell type compositions across tissue locations.

## Supplementary Information

**Additional file 1: Table S1.** Summary of sparsity across 20 spatial transcriptomics datasets used in the present study.

**Additional file 2: Table S2.** Summary of likelihood ratio test results across datasets.

**Additional file 3: Table S3.** scRNA data used as reference for deconvolution in CARD.

**Additional file 4: Table S4.** Mathematical formula for the estimated mean, variance and zero proportion in the four count models that include the Poisson, negative binomial, zero-inflated Poisson and zero-inflated negative binomial models.

**Additional file 5: Table S5.** Location clustering information for each dataset.

**Additional file 6: Table S6.** Database of individual datasets and accompanying citations used throughout this study.

**Additional file 7: Figure S1.** The relationship between the zero proportion and the mean across genes are displayed for datasets not shown in Fig. 1. **Figure S2.** The relationship between the variance and the mean is displayed across genes for datasets not shown in Fig. 2. **Figure S3.** Accounting for cell type mixtures reveals shifted count model preference and substantially reduced overdispersion across datasets. **Figure S4.** The average AIC across genes and datasets for each of the four count models with an offset is displayed against that without an offset. **Figure S5.** UMAP plot shows the location clustering pattern for 15 datasets.

**Additional file 8.** Review history.

## Data Availability

Repository link for individual datasets can be found in Additional file 6: Table S6. The R code (license: Creative Commons Attribution 3.0 United States) and data for reproducing the results in Figs. 1, 2, 3, and 4 and Additional file 7 is available at 10.5281/zenodo.6503597 [57]. Code availability: Scripts for reproducing all results in the present study are also available under MIT license at Github https://github.com/Peiyao-Z/zero-count-analysis [58].

## References

[1] Ji N, van Oudenaarden A (2012). Single molecule fluorescent in situ hybridization (smFISH) of C. elegans worms and embryos.

[2] Rahman S, Zenklusen D (2013). Single-molecule resolution fluorescent in situ hybridization (smFISH) in the yeast *S. cerevisiae*. Methods Mol Biol.

[3] Wang S (2019). Single molecule RNA FISH (smFISH) in whole-mount mouse embryonic organs. Curr Protoc Cell Biol.

[4] Eng C-HL (2019). Transcriptome-scale super-resolved imaging in tissues by RNA seqFISH+. Nature.

[5] Shah S (2016). In situ transcription profiling of single cells reveals spatial organization of cells in the mouse hippocampus. Neuron.

[6] Chen KH (2015). Spatially resolved, highly multiplexed RNA profiling in single cells. Science.

[7] Moffitt JR (2016). High-throughput single-cell gene-expression profiling with multiplexed error-robust fluorescence in situ hybridization. Proc Natl Acad Sci.

[8] Bidarimath M, Edwards AK, Tayade C (2015). Laser capture microdissection for gene expression analysis. Methods Mol Biol.

[9] Nakamura T (2004). Genome-wide cDNA microarray analysis of gene expression profiles in pancreatic cancers using populations of tumor cells and normal ductal epithelial cells selected for purity by laser microdissection. Oncogene.

[10] Nichterwitz S (2016). Laser capture microscopy coupled with Smart-seq2 for precise spatial transcriptomic profiling. Nat Commun.

[11] Kruse F (2016). Tomo-seq: A method to obtain genome-wide expression data with spatial resolution. Methods Cell Biol.

[12] Lubeck E, Cai L (2012). Single-cell systems biology by super-resolution imaging and combinatorial labeling. Nat Methods.

[13] Ståhl PL (2016). Visualization and analysis of gene expression in tissue sections by spatial transcriptomics. Science.

[14] Rodriques SG (2019). Slide-seq: a scalable technology for measuring genome-wide expression at high spatial resolution. Science.

[15] Stickels RR (2021). Highly sensitive spatial transcriptomics at near-cellular resolution with Slide-seqV2. Nat Biotechnol.

[16] Vickovic S (2019). High-definition spatial transcriptomics for in situ tissue profiling. Nat Methods.

[17] Cho CS (2021). Microscopic examination of spatial transcriptome using Seq-Scope. Cell.

[18] Sun S, Zhu J, Zhou X (2020). Statistical analysis of spatial expression patterns for spatially resolved transcriptomic studies. Nat Methods.

[19] Andersson A (2020). Single-cell and spatial transcriptomics enables probabilistic inference of cell type topography. Commun Biol.

[20] Cable DM, Murray E, Zou LS, et al. Robust decomposition of cell type mixtures in spatial transcriptomics. Nat Biotechnol. 2022;40:517–26. 10.1038/s41587-021-00830-w. 10.1038/s41587-021-00830-w PMC8606190 33603203

[21] Lopez R (2019). A joint model of unpaired data from scRNA-seq and spatial transcriptomics for imputing missing gene expression measurements.

[22] BinTayyash N, Georgaka S, John ST, et al. Non-parametric modelling of temporal and spatial counts data from RNA-seq experiments [published online ahead of print, 2021 Jul 2]. Bioinformatics. 2021;btab486. 10.1093/bioinformatics/btab486. 10.1093/bioinformatics/btab486 PMC10186154 34213536

[23] Cho H, et al. A bivariate zero-inflated negative binomial model and its applications to biomedical settings. bioRxiv. 2021. p. 2020.03.06.977728. 10.1177/09622802231172028 PMC10500952 37167422

[24] Jiang R (2022). Statistics or biology: the zero-inflation controversy about scRNA-seq data. Genome Biol.

[25] Kim TH, Zhou X, Chen M (2020). Demystifying “drop-outs” in single-cell UMI data. Genome Biol.

[26] Svensson V (2020). Droplet scRNA-seq is not zero-inflated. Nat Biotechnol.

[27] Sarkar A, Stephens M (2021). Separating measurement and expression models clarifies confusion in single-cell RNA sequencing analysis. Nat Genet.

[28] Bergenstråhle L, He B, Bergenstråhle J (2022). Super-resolved spatial transcriptomics by deep data fusion. Nat Biotechnol..

[29] Satija R, Farrell JA, Gennert D, Schier AF, Regev A (2015). Spatial reconstruction of single-cell gene expression data. Nat Biotechnol..

[30] Korsunsky I (2019). Fast, sensitive and accurate integration of single-cell data with Harmony. Nat Methods.

[31] Arisdakessian C (2019). DeepImpute: an accurate, fast, and scalable deep neural network method to impute single-cell RNA-seq data. Genome Biol.

[32] Ma Y, Zhou X. Spatially informed cell-type deconvolution for spatial transcriptomics [published online ahead of print, 2022 May 2]. Nat Biotechnol. 2022:10.1038/s41587-022-01273-7. 10.1038/s41587-022-01273-7. 10.1038/s41587-022-01273-7 PMC9464662 35501392

[33] Zhu J, Sun S, Zhou X (2021). SPARK-X: non-parametric modeling enables scalable and robust detection of spatial expression patterns for large spatial transcriptomic studies. Genome Biol.

[34] Svensson V, Teichmann SA, Stegle O (2018). SpatialDE: identification of spatially variable genes. Nat Methods.

[35] Human Breast Cancer (Block A Section 1), Spatial Gene Expression Dataset by Space Ranger 1.1.0, 10x Genomics, (2020, June 23).

[36] Human Heart, Spatial Gene Expression Dataset by Space Ranger 1.1.0, 10x Genomics, (2020, June 23).

[37] Human Lymph Node, Spatial Gene Expression Dataset by Space Ranger 1.1.0, 10x Genomics, (2020, June 23).

[38] Mouse Kidney Section (Coronal), Spatial Gene Expression Dataset by Space Ranger 1.1.0, 10x Genomics, (2020, June 23).

[39] Mouse Brain Section (Coronal), Spatial Gene Expression Dataset by Space Ranger 1.1.0, 10x Genomics, (2020, June 23).

[40] Mouse Brain Serial Section 1 (Sagittal-Anterior), Spatial Gene Expression Dataset by Space Ranger 1.1.0, 10x Genomics, (2020, June 23).

[41] Mouse Brain Serial Section 1 (Sagittal-Posterior), Spatial Gene Expression Dataset by Space Ranger 1.1.0, 10x Genomics, (2020, June 23).

[42] Wang X (2018). Three-dimensional intact-tissue sequencing of single-cell transcriptional states. Science.

[43] Moor AE (2018). Spatial reconstruction of single enterocytes uncovers broad zonation along the intestinal villus axis. Cell.

[44] Baccin C (2020). Combined single-cell and spatial transcriptomics reveal the molecular, cellular and spatial bone marrow niche organization. Nat Cell Biol.

[45] Halpern KB (2018). Paired-cell sequencing enables spatial gene expression mapping of liver endothelial cells. Nat Biotechnol.

[46] Team RC (2020). R: A Language and Environment for Statistical Computing.

[47] Venables WN, RB (2002). Modern Applied Statistics with S. Fourth ed.

[48] Jackman S (2020). {pscl}: Classes and methods for {R} developed in the Political Science Computational Laboratory.

[49] Zeileis A, Kleiber C, Jackman S (2008). Regression models for count data in R. J Stat Softw.

[50] Bates D, Maechler M (2021). Matrix: sparse and dense matrix classes and methods.

[51] Hao Y, Hao S, Andersen-Nissen E (2021). Integrated analysis of multimodal single-cell data. Cell..

[52] Azizi E (2018). Single-cell map of diverse immune phenotypes in the breast tumor microenvironment. Cell.

[53] Asp M (2019). A spatiotemporal organ-wide gene expression and cell atlas of the developing human heart. Cell.

[54] Zeisel A (2018). Molecular architecture of the mouse nervous system. Cell.

[55] Park J (2018). Single-cell transcriptomics of the mouse kidney reveals potential cellular targets of kidney disease. Science.

[56] Tepe B (2018). Single-cell RNA-seq of mouse olfactory bulb reveals cellular heterogeneity and activity-dependent molecular census of adult-born neurons. Cell Rep.

[57] Zhao P, Zhu J, Ma Y, Zhou X. Modeling zero inflation is not necessary for spatial transcriptomics. Zenodo. 2022. 10.5281/zenodo.6503597. 10.1186/s13059-022-02684-0 PMC9116027 35585605

[58] Zhao P, Zhu J, Ma Y, Zhou X (2022). Modeling zero inflation is not necessary for spatial transcriptomics.

