# Supplementary material for: Modeling zero inflation is not necessary for spatial transcriptomics
Source: Genome Biol. 2022 May 18;23:118. doi: 10.1186/s13059-022-02684-0 (PMC9116027; doi:10.1186/s13059-022-02684-0)
Supplement: Supplementary file 7 — Additional file 7: Figure S1. The relationship between the zero proportion and the mean across genes are displayed for datasets not shown in Fig. 1. Figure S2. The relationship between the variance and the mean is displayed across genes for datasets not shown in Fig. 2. Figure S3. Accounting for cell type mixtures reveals shifted count model preference and substantially reduced overdispersion across datasets. Figure S4. The average AIC across genes and datasets for each of the four count models with an offset is displayed against that without an offset. Figure S5. UMAP plot shows the location clustering pattern for 15 datasets. [file 13059_2022_2684_MOESM7_ESM.docx]

**Figure S1 The relationship between the zero proportion and the mean across genes are displayed for datasets not shown in Figure 1.**

**

**The zero proportion versus mean trend is fitted by either a Poisson model (blue line) or a negative binomial model (red line) across genes.

**Figure S2 The relationship between the variance and the mean is displayed across genes for datasets not shown in Figure 2.**

**
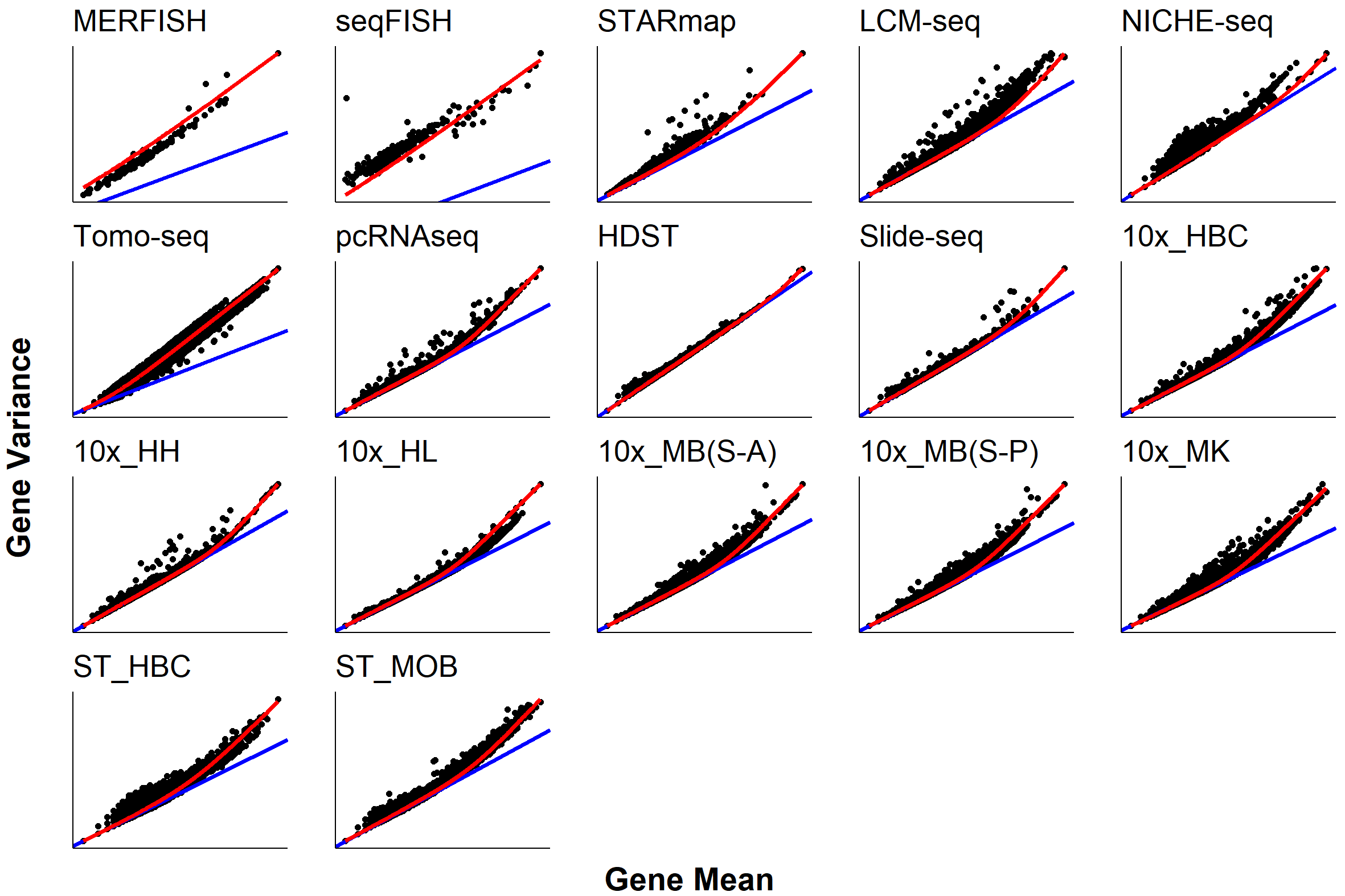
**The variance versus mean trend is fitted by either a Poisson model (blue line) or a negative binomial model (red line) across genes.

**Figure S3: Accounting for cell type mixtures reveals shifted count model preference and substantially reduced over dispersion across datasets.**


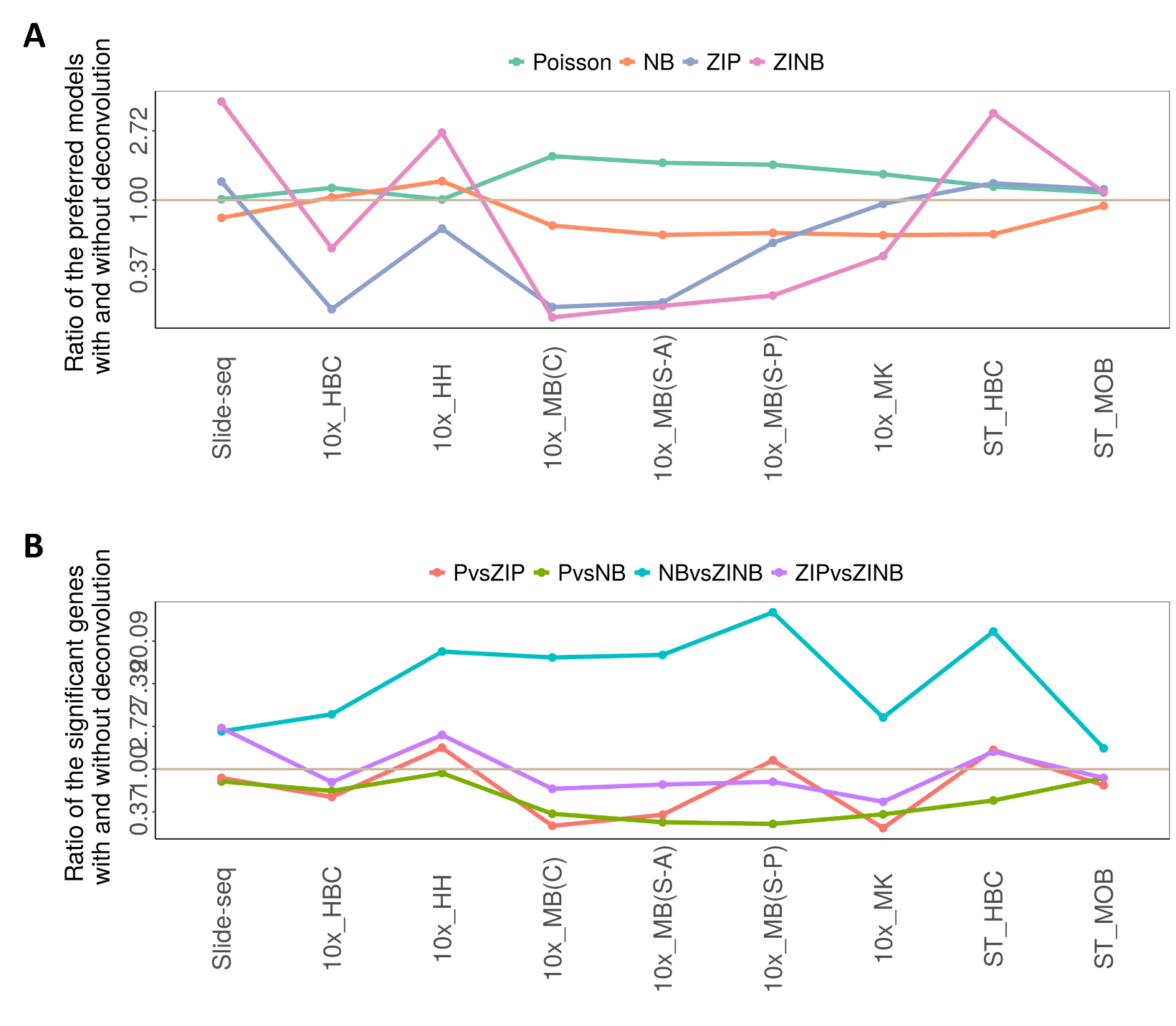
**A:** Ratio of proportion of genes that prefer each of the four count models based on model selection with AIC (y-axis) before and after accounting for cell type mixtures is displayed for each dataset (x-axis). The four models include Poisson (Aquamarine), negative binomial (NB; Tangerine), zero-inflated Poisson (ZIP; Polo Blue), and zero-inflated negative binomial (ZINB; pink). **B**: Ratio of proportion of genes that are significant in each of the four likelihood ratio tests (y-axis) before and after accounting for cell type mixtures is displayed for each dataset (x-axis). The four LRT tests include the test on P vs ZIP (salmon), P vs NB (green), NB vs ZINB (cyan), and ZIP vs ZINB (purple).

**Figure S4 The average AIC across genes and datasets for each of the four count models with an offset is displayed against that without an offset.**

**
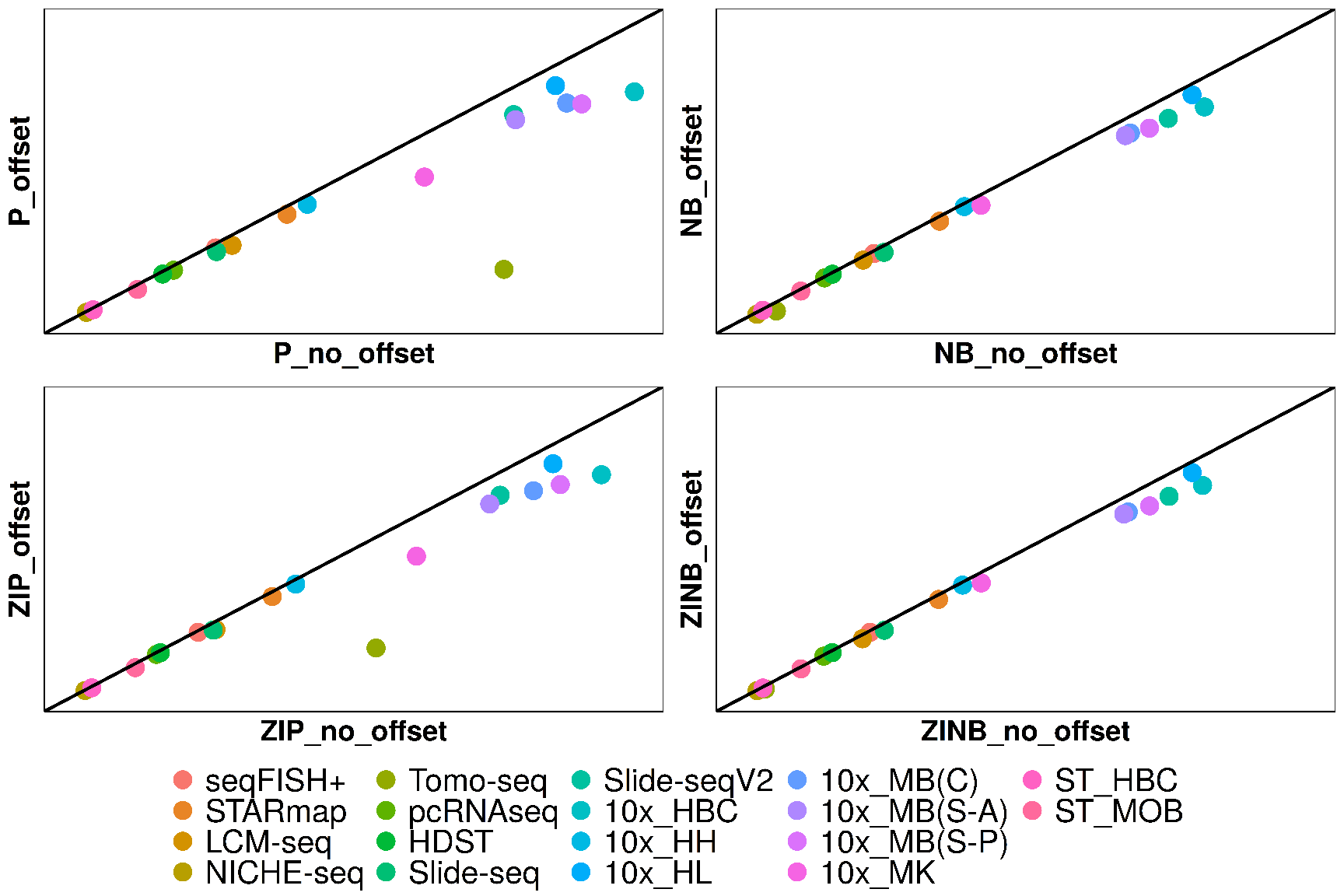
**The solid line is the diagonal line. For better visualization, we excluded MERFISH and seqFISH data that their AICs of each model with offset are much larger than those with no offset.

**Figure S5 UMAP plot shows the location clustering pattern for 15 datasets.**

**
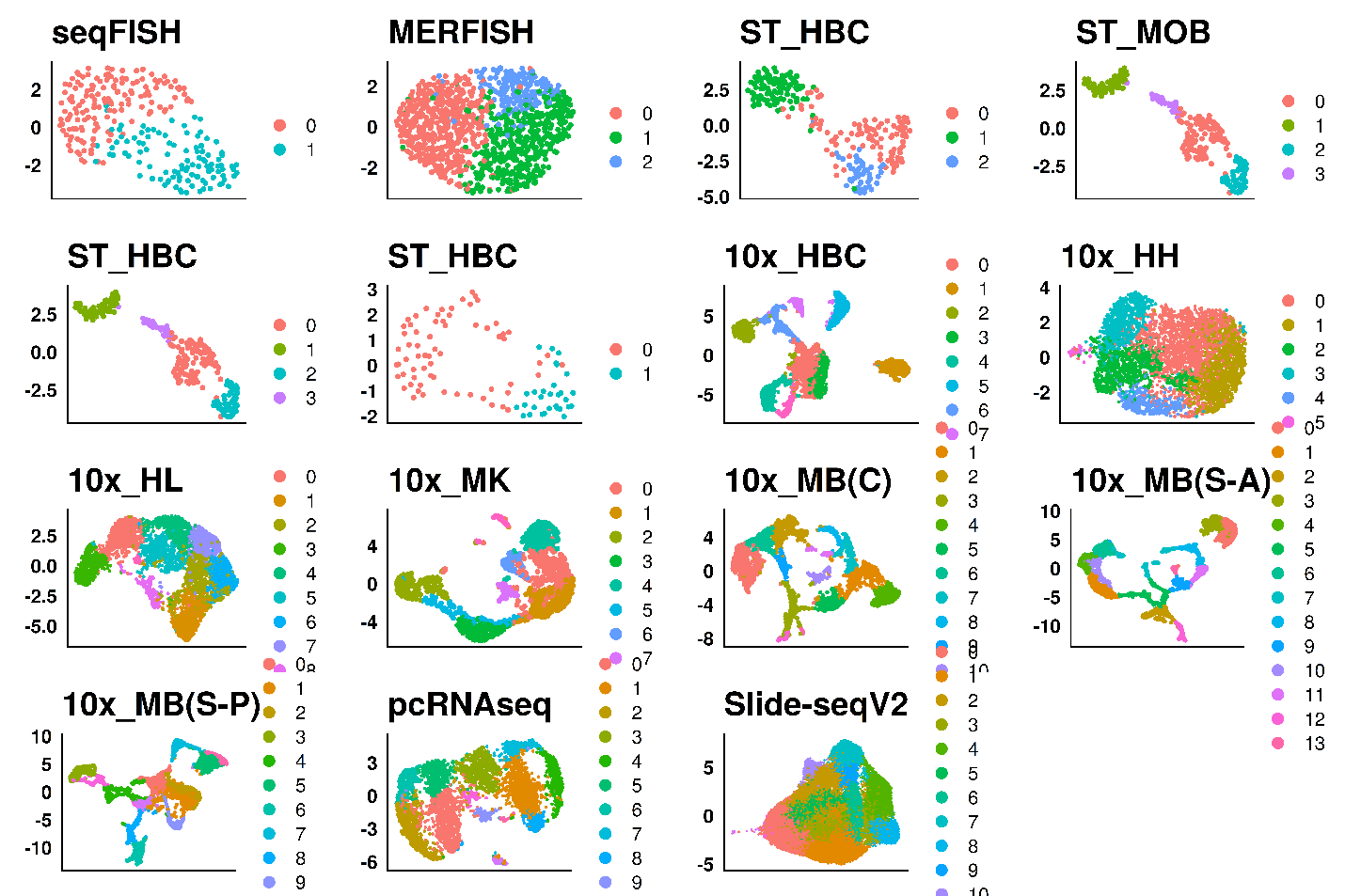
**
