# Supplementary material for: Modeling zero inflation is not necessary for spatial transcriptomics
Source: Genome Biol. 2022 May 18;23:118. doi: 10.1186/s13059-022-02684-0 (PMC9116027; doi:10.1186/s13059-022-02684-0)
Supplement: Supplementary file 8 — Additional file 8. Review history. [file 13059_2022_2684_MOESM8_ESM.docx]

Review History

**First round of review**

**Reviewer 1**

**Were you able to assess all statistics in the manuscript, including the appropriateness of statistical tests used?**

Yes, and I have assessed the statistics in my report.

**Comments to author:**

In this paper, the authors collected 20 spatial transcriptomics datasets that were generated by 11 different technology platforms, and examined the distribution of zeros in those datasets. For each datasets, the authors performed cross-gene analysis, gene-specific analysis, as well as location/cluster specific analysis, with multiple statistical methods. The methods are relatively straightforward, and are close parallels to several previous papers that examined the same question on scRNA-seq data. The conclusion is also parallel with previous results in scRNA-seq, showing that the zeros can be largely explained by the heterogeneity among cells/locations, and therefore modeling zeros inflation is not necessary. The results in this paper provides convincing support to the conclusion.

Just one minor comment, in the middle of the abstract, "with a considerable overlap between the two gene sets", it does not read well. Is it a typo and should be two datasets? Or, if not, what are the gene sets and how are they defined?

**Reviewer 2**

**Were you able to assess all statistics in the manuscript, including the appropriateness of statistical tests used?**

Yes, and I have assessed the statistics in my report.

**Comments to author:**

Zhao et al. reported findings from a systematic evaluation of the statistical distribution for gene counts from spatial transcriptomics studies. The authors analyzed 20 different datasets collected from 11 distinct technologies. Through comprehensive analyses, they characterized the distributional properties of gene counts by examining which statistical distribution fits the best for the data. They examined 4 distributions: Poisson, Negative Binomial, Zero inflated Poisson and Zero inflated Negative Binomial. The results from their analyses indicate that there is no need to model zero inflation because for most genes Poisson and Negative Binomial are sufficient. Furthermore, they find that the overdispersion can be explained by location heterogeneity, and when restricting analyses to relatively homogeneous regions, Poisson model is often sufficient. This paper is the first comprehensive study on the statistical distributions of gene count data in spatial transcriptomics. The paper is well-written and the messages are clear and have important implications for statistical modeling of spatial transcriptomics data.

I only have one comment that hopes the authors could address: Some of the sequencing-based spatial transcriptomics technologies, e.g., 10x Visium do not have single-cell resolution. Will the degree of overdispersion decrease even further if you account for cell type mixtures within a spot? Can you include cell type proportions estimated from cell type deconvolution analyses to evaluate how much the degree of overdispersion is decreased?

**Authors Response**

**Point-by-point responses to the reviewers’ comments:**

Reviewer: 1#

General Comments:

In this paper, the authors collected 20 spatial transcriptomics datasets that were generated by 11 different technology platforms, and examined the distribution of zeros in those datasets. For each datasets, the authors performed cross-gene analysis, gene-specific analysis, as well as location/cluster specific analysis, with multiple statistical methods. The methods are relatively straightforward, and are close parallels to several previous papers that examined the same question on scRNA-seq data. The conclusion is also parallel with previous results in scRNA-seq, showing that the zeros can be largely explained by the heterogeneity among cells/locations, and therefore modeling zeros inflation is not necessary. The results in this paper provides convincing support to the conclusion.

*Response: Thank you for the positive comments and constructive suggestions. Our detailed response to your specific comment is listed below.*

Specific Comments:

Just one minor comment, in the middle of the abstract, "with a considerable overlap between the two gene sets", it does not read well. Is it a typo and should be two datasets? Or, if not, what are the gene sets and how are they defined?

*Response: Thank you. We have clarified this by modifying the corresponding sentence to “with genes displaying over dispersion substantially overlapped with genes displaying zero inflation.” (lines 22-23 on page 1).*

Reviewer: 2#

General Comments:

Zhao et al. reported findings from a systematic evaluation of the statistical distribution for gene counts from spatial transcriptomics studies. The authors analyzed 20 different datasets collected from 11 distinct technologies. Through comprehensive analyses, they characterized the distributional properties of gene counts by examining which statistical distribution fits the best for the data. They examined 4 distributions: Poisson, Negative Binomial, Zero inflated Poisson and Zero inflated Negative Binomial. The results from their analyses indicate that there is no need to model zero inflation because for most genes Poisson and Negative Binomial are sufficient. Furthermore, they find that the overdispersion can be explained by location heterogeneity, and when restricting analyses to relatively homogeneous regions, Poisson model is often sufficient. This paper is the first comprehensive study on the statistical distributions of gene count data in spatial transcriptomics. The paper is well-written and the messages are clear and have important implications for statistical modeling of spatial transcriptomics data.

*Response: Thank you for the positive comments and constructive suggestions. Our detailed response to your specific comment is listed below.*

Specific Comments:

I only have one comment that hopes the authors could address: Some of the sequencing-based spatial transcriptomics technologies, e.g., 10x Visium do not have single-cell resolution. Will the degree of overdispersion decrease even further if you account for cell type mixtures within a spot? Can you include cell type proportions estimated from cell type deconvolution analyses to evaluate how much the degree of overdispersion is decreased?

*Response: Thank you. Follow your suggestion, we have included an additional analysis to examine whether the degree of over-dispersion decrease after controlling for cell type composition. To do so, we focused on nine spatial transcriptomics datasets where we were able to obtain a published single cell RNA sequencing reference data measured on the same tissue. On each of the nine datasets, we performed reference-based cell type deconvolution using CARD to infer the cell type composition on each tissue location. Afterwards, we carried out conditional analysis where we controlled for the estimated cell type compositions by including them as covariates. In the analysis, we found that the fraction of genes preferring the Poisson model after controlling for cell type compositions substantially increases while proportion of over-dispersed genes after controlling for cell type compositions substantially reduced, supporting that the degree of over dispersion indeed substantially decreases after controlling for cell type compositions. These details are provided in the updated Results section (lines 277-297 on pages 7-8).*
